# Viral Infection and the Blood-Brain Barrier: Molecular Research Insights and Therapies

**DOI:** 10.1093/infdis/jiaf455

**Published:** 2025-09-29

**Authors:** Sarah A Boardman, Claire Hetherington, Thomas Hughes, Callum Cook, Ian Galea, Orla Hilton, Tom Solomon, Andrew D Luster, Stuart Allan, Evelyn Kurt-Jones, Joe Forth, Adjanie Patabendige, Franklyn N Egbe, Cordelia Dunai, Benedict D Michael

**Affiliations:** Institute of Infection, Veterinary and Ecological Science, Clinical Infection, Microbiology, and Immunology, University of Liverpool, Liverpool, United Kingdom; Institute of Infection, Veterinary and Ecological Science, Clinical Infection, Microbiology, and Immunology, University of Liverpool, Liverpool, United Kingdom; Institute of Infection, Veterinary and Ecological Science, Clinical Infection, Microbiology, and Immunology, University of Liverpool, Liverpool, United Kingdom; Institute of Infection, Veterinary and Ecological Science, Clinical Infection, Microbiology, and Immunology, University of Liverpool, Liverpool, United Kingdom; Clinical Neurosciences, Clinical and Experimental Sciences, Faculty of Medicine, University of Southampton, Southampton, United Kingdom; Institute of Infection, Veterinary and Ecological Science, Clinical Infection, Microbiology, and Immunology, University of Liverpool, Liverpool, United Kingdom; Institute of Infection, Veterinary and Ecological Science, Clinical Infection, Microbiology, and Immunology, University of Liverpool, Liverpool, United Kingdom; NIHR Health Protection Research Unit in Emerging and Zoonotic Infections, University of Liverpool, Liverpool, United Kingdom; The Walton Centre National Health Service (NHS) Foundation Trust, Liverpool, United Kingdom; The Pandemic Institute, Liverpool, United Kingdom; Center for Immunology and Inflammatory Diseases, Division of Rheumatology, Allergy and Immunology, Massachusetts General Hospital, Harvard Medical School, Charlestown, Massachusetts, USA; Division of Neuroscience, School of Biological Sciences, Faculty of Biology, Medicine and Health, University of Manchester, Manchester, United Kingdom; Geoffrey Jefferson Brain Research Centre, Manchester Academic Health Science Centre, Northern Care Alliance NHS Foundation Trust, University of Manchester, Manchester, United Kingdom; Division of Infectious Diseases and Immunology, Department of Medicine, University of Massachusetts Chan Medical School, Worcester, USA; Department of Chemistry, University of Liverpool, Liverpool, United Kingdom; Department of Physics, University of Liverpool, Liverpool, United Kingdom; Institute of Infection, Veterinary and Ecological Science, Clinical Infection, Microbiology, and Immunology, University of Liverpool, Liverpool, United Kingdom; College of Science and Engineering, University of Derby, Derby, United Kingdom; Liverpool Centre for Cardiovascular Science, University of Liverpool, United Kingdom; Institute of Infection, Veterinary and Ecological Science, Clinical Infection, Microbiology, and Immunology, University of Liverpool, Liverpool, United Kingdom; Institute of Infection, Veterinary and Ecological Science, Clinical Infection, Microbiology, and Immunology, University of Liverpool, Liverpool, United Kingdom; NIHR Health Protection Research Unit in Emerging and Zoonotic Infections, University of Liverpool, Liverpool, United Kingdom; Institute of Infection, Veterinary and Ecological Science, Clinical Infection, Microbiology, and Immunology, University of Liverpool, Liverpool, United Kingdom; NIHR Health Protection Research Unit in Emerging and Zoonotic Infections, University of Liverpool, Liverpool, United Kingdom; The Walton Centre National Health Service (NHS) Foundation Trust, Liverpool, United Kingdom

**Keywords:** blood-brain barrier, mechanisms, virology

## Abstract

The blood-brain barrier (BBB) protects the brain from pathogenic microorganisms. Neurologic complications from viral infections, including herpes simplex virus, varicella zoster virus, HIV, Japanese encephalitis virus, and SARS-CoV-2, are linked to BBB dysfunction and loss of barrier integrity. Increased BBB permeability associated with viral infections can occur through several mechanisms, such as direct neurotropism, Trojan horse mechanisms, or systemic infection and inflammation. Viruses cause direct and indirect immune-mediated damage. Understanding these neuroimmune mechanisms is critical to establish therapeutic strategies to protect BBB function. This review describes the effect of viral infection on the BBB, clinical methods to assess BBB integrity, and clinical management approaches to address viral-induced BBB damage.

## Teaser

Viral infections can disrupt the blood-brain barrier via direct and indirect mechanisms and cause neurologic complications. Current treatment is limited to antivirals and broad immune suppression to control the infection and inflammation. There is recent focus on more targeted adjunctive therapies.

**Figure 1.**
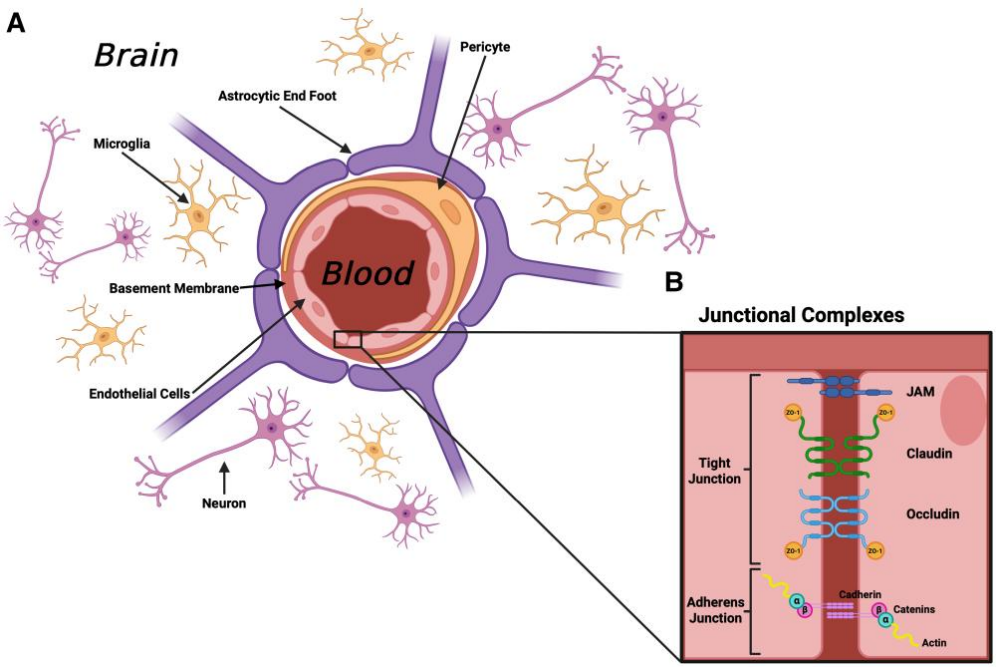
Schematic of the blood-brain barrier and neurovascular unit, displaying junctional complexes. *A*, Endothelial cells line the lumen of blood vessels, partially encircled by pericytes embedded in a basement membrane. Astrocytic foot processes support the outer perimeter of the lumen. The wider neurovascular unit contains the resident immune cells of the central nervous system, microglia, and neurons. *B*, Endothelial cells are tightly adhered by junctional complexes known as *tight junctions* and *adherent junctions*, which strengthen the structural integrity of the endothelial monolayer. JAM, junctional adhesion molecule; ZO, zonula occludens.

The cells that form the neurovascular unit (NVU) of the central nervous system (CNS) are critical for maintaining the blood-brain barrier (BBB) through the regulation of molecules, ions, and pathogens transiting from the bloodstream [1]. The key components of the BBB are brain endothelial cells, pericytes, and astrocytes (Figure 1*A*). Brain endothelial cells are the most important component of the BBB and exhibit specific phenotypically distinct characteristics to reduce paracellular transcytosis [1]. This includes tight junctions (TJs) and adherent junctions between adjoining endothelial cells (Figure 1*B*). Astrocytes extend endfeet projections that encase brain endothelial cells, pericytes, and the basement membrane to provide support to the BBB and maintain its integrity. Pericytes are also important in the structural maintenance and regulation of the BBB. Adjacent cells in the extravascular regions of the brain include neurons and the resident CNS immune cells, microglia, which form the broader NVU. Each component (supplementary appendix) ensures normal physiologic function, and when damaged or altered, the barrier becomes dysfunctional and loses integrity. Viral infections can cause BBB breakdown and severe neurologic manifestations, including encephalitis [2], meningitis [3], and microcephaly [4], risking significant neurologic morbidity and mortality [5, 6]. An improved understanding of the underlying mechanistic interactions driving BBB and NVU breakdown is critical to repurpose or develop neuroprotective therapies to reduce cellular injury and cerebral edema and thus minimize sequelae [7]. For most viral infections affecting the CNS, current therapeutic options primarily address pathogen replication while fewer are currently available to specifically address BBB damage.

## BIOMARKERS OF BBB INTEGRITY

The BBB can be assessed clinically by utilizing biomarkers of solute influx or efflux or damage to the NVU structure.

### Systemic Protein Influx

As albumin is not synthesized in significant concentration in the CNS, the ratio between cerebrospinal fluid (CSF) and serum albumin concentration (CSF/serum albumin quotient) is often used as a BBB permeability index [8]. However, as activated microglia may produce albumin [9], this may confound measurement, particularly during viral infection. Since CSF is produced by the choroid plexus, another limitation of the CSF/serum albumin quotient is that the CSF albumin concentration may not directly reflect the brain's interstitial fluid. Anatomically and physiologically, it is important to note that the blood-brain interstitial fluid and blood-CSF are distinct. The blood-CSF barrier is considered more permeable due to consisting of epithelial cells of the choroid plexus with less restrictive TJs [10]. A more accurate and minimally invasive approach is dynamic contrast–enhanced magnetic resonance imaging (MRI) [11]. By imaging the brain serially before, during, and after paramagnetic gadolinium-containing contrast agent injection, signal change within the brain tissue is converted to gadolinium concentration and the transfer coefficient calculated through Patlak modeling of the tissue concentration–time curve [12].

### Systemic Protein Efflux

Several proteins are highly expressed in the brain, but their concentration is extremely low or absent in blood under healthy conditions; therefore, they can be used as blood biomarkers. During viral infection, the circulating concentration is also influenced by the severity of neuropathology. Nevertheless, they can still function as BBB permeability markers if statistical analyses are appropriately controlled for disease severity. Examples of commonly studied markers include glial fibrillary acidic protein (GFAP) and S100B of astrocytic origin, as well as ubiquitin C-terminal hydrolase L1, neurofilament light (NfL), and tau, which are neuronally derived [13]. Due to their low concentrations in serum and plasma, even during BBB breakdown, ultrasensitive methods with technology such as single-molecule array are required [14].

### NVU Damage

Release of molecular or subcellular components of the NVU during BBB damage provides a source of biomarkers in CSF and blood. While serum levels of fragments or soluble forms of cellular adhesion molecules correlate with BBB damage, these are not specific to the cerebral vasculature [15]. It is important to note that peripheral nerve damage can cause the release of NfL and therefore reflect peripheral nervous system and CNS injury [16]. More recent studies have utilized molecules that are highly expressed at the BBB, such as CSF-soluble platelet-derived growth factor receptor β [17] or extracellular vesicles positive for cerebral endothelial markers by flow cytometry [18].

## VIRAL INFECTIONS THAT CAUSE DISRUPTION TO THE BBB

Whether from direct infection or indirect effects (ie, immune-mediated pathology), viral infection can cause major complications, such as encephalitis, meningitis, microencephaly, and demyelination. While a wide spectrum of viruses can induce CNS injury and BBB breakdown, this review focuses on those of greatest human burden.

### Herpes Simplex Virus

Herpes simplex virus 1 (HSV-1) is the most common cause of sporadic viral encephalitis in adults and children in high-income settings [19]. Herpes simplex encephalitis (HSE) has an estimated incidence of 1 of 100 000 to 150 000 people per year [20]. Typically, patients present with a combination of new headaches, fever or febrile/coryzal prodrome, and nausea during the early stages of encephalitis. As the disease progresses, typically over hours or days, patients develop further symptoms, including alterations in cognition, consciousness, personality, and/or behavior, and a proportion will develop focal neurologic signs and seizures [21]. The majority of people have been infected by HSV-1 by the time that they reach adulthood. HSV-1 is transmitted by droplet spread, crosses the oral and/or nasal mucosa, enters sensory neurons, and migrates by retrograde axonal transport to the trigeminal ganglia, where it establishes latency, although the virus may also migrate via the olfactory bulb [22]. During primary infection and following reactivation from latency, CNS infection with HSV-1 occurs by further retrograde axonal transport to establish infection, predominantly affecting the temporal lobes and orbitofrontal cortex, often bilaterally but asymmetrically, or less commonly the brainstem. Active HSV replication within the CNS induces an immune response that drives the recruitment of neutrophils and monocytes across the BBB and increased permeability of the BBB with vasogenic edema [7]. Much of the inflammatory response to HSV-1 infection in the CNS is due to the localized production of cytokines and chemokines. Abrogation of CXCL1 signaling, through either the administration of neutralizing antibodies or the absence of the cognate receptor CXCR2, reduces neutrophil recruitment into the CNS and diminishes BBB permeabilization [7]. Microglia produce a robust cytokine and chemokine response after HSV-1 infection in vitro [23]. Importantly, CXCL1 is also produced by uninfected perivascular astrocytes in response to paracrine production of IL-1 and by microglia responding to pathogen- and damage-associated molecular patterns produced by HSV-1–infected neurons. In human disease, the proinflammatory effects of the IL-1 cytokine family are balanced by expression of the anti-inflammatory endogenous IL-1 receptor antagonist (eg, IL-1Ra). This pro- vs anti-inflammatory balance is associated with changes in BBB permeability, as measured by albumin leakage into the CSF and edema on MRI, and correlates with clinical disease severity and poor outcome [24].

### Varicella Zoster Virus

Varicella zoster virus (VZV) causes chickenpox during primary infection and shingles following reactivation of latent infection. Similarly to HSV-1, VZV can remain latent in neurons but typically is found in the dorsal root ganglia of spinal sensory neurons [25]. Post–varicella zoster complications occur as a result of reactivation of the virus and often affect those who are elderly or immunocompromised. Most commonly, this manifests as postherpetic neuralgia but can result in other neurologic complications, including encephalitis, meningitis, myelitis, and inflammatory vasculopathies affecting the small and/or large blood vessels of the CNS [26]. Other viruses that directly infect brain endothelial cells include henipaviruses: Nipah virus and Hendra virus [27]. These VZV-associated vascular events can drive further complications, such as ischemic and hemorrhagic stroke [26]. Collection of CSF and serum from patients positive for VZV CNS infection showed an increase in the levels of proinflammatory chemokines, including CCL19, CXCL8, CXCL9, and CXCL10, and matrix metalloproteinases (MMPs), such as MMP-2, MMP-3, MMP-8, MMP-9, and MMP-12 [28]. MMPs have been associated with BBB breakdown by causing degradation of the basement membrane, detachment of astrocytes, and cleavage of TJ proteins [29]. Infection of different cerebrovascular cells with VZV was shown to increase the expression of proinflammatory cytokines, especially IL-8 and IL-6 [30]. Another study found that treating human neurons with IL-6 reduced viral replication of VZV in vitro [31]. However, there is a lack of research revealing the cellular mechanistic interactions and the impact on the BBB in CNS infections with VZV.

### Japanese Encephalitis Virus

Japanese encephalitis virus (JEV) is the predominant cause of viral encephalitis in Asia. Other neuropathic flaviviruses include West Nile virus, dengue virus, yellow fever virus, and Zika virus [32]. JEV disrupts BBB function and causes >67 000 cases of encephalitis annually [33]. Symptoms begin with the development of fever and progress to a decline in consciousness, vomiting, headaches, seizures, and sometimes focal movement disorders, reflecting involvement of the basal ganglia [34]. Multiple routes of entry of JEV into the CNS have been proposed, including the “Trojan horse” mechanism utilizing infected immune cells or as a result of BBB disruption from systemic infection. One study based on an in vitro Transwell BBB model found that JEV, even at low titers (multiplicity of infection = 1), was able to infect endothelial cells, which aided in viral replication [35]. JEV replication caused an increase in BBB permeability due to activation of the endothelium and release of proinflammatory mediators, allowing virus to cross into the brain. Alternative suggestions propose that JEV initially enters the CNS prior to disrupting the BBB and promotes an immune response, which causes subsequent BBB damage and promotes further viral entry [36].

Production of inflammatory chemokines (eg, CXCL10) secreted from JEV-infected glial cells leads to further damage of NVU components and increased BBB permeability [36, 37]. In mice infected with JEV, attenuation of CXCL10 prevents the decrease in TJ expression and restores endothelial integrity. In vitro studies have reported upregulation of CXCR3 (receptor for CXCL10) in astrocytes during JEV infection. CXCL10/CXCR3 binding triggers the production of TNF and significantly decreases the expression of occludin, claudin 5, and ZO-1, thus promoting BBB damage [37]. Microglial activation is also induced by the secretion of proinflammatory cytokines from JEV-infected neurons [38]. This cytokine response is initially beneficial for immune cell recruitment and viral clearance, but prolonged activation of microglia causes overproduction of proinflammatory factors that contribute to neuronal injury and death [39].

### HIV

HIV, when untreated, can advance to become AIDS. The World Health Organization estimates that there are 39 million people with HIV worldwide and 360 000 deaths per year [40]. Initial HIV infection can result in a clinical presentation of encephalitis during seroconversion, but even without encephalitis symptoms, it is established that HIV enters the CNS early during infection [41, 42]. Subcortical white matter changes are a specific characteristic of HIV-related CNS complications. BBB damage is thought to contribute to these changes as well as the development of further neurologic complications, such as AIDS dementia [43]. Later, HIV can cause acute CD8^+^ T-cell encephalitis or more subtle neurologic complications, which have been termed *HIV-associated neurocognitive disorders*, referring to deficits in cognition, concentration, and motor skills [44]. HIV can cross the BBB by using a Trojan horse mechanism via infected macrophages or as free virions entering through endothelial cells. HIV-1 can enter macrophages by targeting CD4 and CCR5 receptors, and these infected cells can then cross the BBB, leading to release of inflammatory cytokines and neuronal injury [45]. Neuronal injury from HIV infection may increase BBB permeability within days [46], with TJ damage found in brain tissue taken from a patient with HIV who developed encephalitis. Fragmented expression or a lack of TJ proteins occludin and ZO-1 was observed by immunohistochemistry but were not found in patients with HIV who did not have encephalitis [47].

### SARS-CoV-2

There is increasing evidence that SARS-CoV-2 can cause dysfunction of the BBB [48]. Nevertheless, from human autopsy studies, it is clear that although the virus can be detected in the brain by quantitative polymerase chain reaction, only a minority of cases show virion protein in the brain parenchyma. This is usually at low levels, indicating the more likely mechanism of the parainfectious effects of SARS-CoV-2 [49, 50]. The concentration of NfL and GFAP in serum correlates with the World Health Organization’s COVID-19 severity scale. Moreover, in people who had acute brain dysfunction (eg, encephalopathy, stroke, encephalitis), NfL, GFAP, and tau were elevated in serum even several months following COVID-19 [51, 52]. BBB damage has been associated with cognitive impairment, often termed *brain fog*, in patients experiencing long COVID. Dynamic contrast–enhanced MRI has identified that patients reporting brain fog may have greater BBB leakage [53]. Additionally, patients with brain fog were found to have elevated S100B, suggesting BBB disruption. Global cognitive deficits were found in patients 1 year after COVID-19 hospitalization [54], which was associated with elevated serum NfL and GFAP. Greater COVID-19 severity may also correlate with brain injury biomarkers [52]. A microfluidic dual lung and BBB chip system incorporating blood mononuclear cells modeled SARS-CoV-2 lung infection and reported more severe BBB injury and neuroinflammation as compared with direct viral exposure. The indirect lung infection model revealed the production of 11 cytokines (eg, IL-6, MCP1, IL-1RA, TNF, CXCL10) by glial cells, which caused further BBB injury [55]. BBB injury was observed in another microfluidic chip model when cerebral microvascular endothelial cells (hCMEC/D3) were exposed to SARS-CoV-2 spike protein [56]. In addition, respiratory support is particularly important for COVID-19 encephalopathy as hypoxic insults and resultant glutamate excitotoxicity can cause BBB breakdown [57]. Moreover, prolonged hypoxia drives production of HIF-1a and downstream activation of MMP-2, which causes BBB breakdown [58, 59].

In vivo modeling of intranasal SARS-CoV-1 infection in ACE2 transgenic mice has shown the use of the olfactory bulb for viral entry into CNS [60]. However, alternative infection routes in animal models are suggested by distinct regions of infection, such as the dorsal vagal complex and substantia nigra. A common symptom of COVID-19 is anosmia [61], raising the possibility of an infection route via the olfactory nerves into the CNS. Yet, evidence of virion presence in the parenchyma of the CNS is limited in the majority of human postmortem studies; therefore, anosmia may reflect local infection outside the CNS [49, 50]. Other studies have evaluated SARS-CoV-2 brain infection by using ACE2 receptors as a pathway for CNS entry (reviewed by Zamorano et al [62]), but the importance of this pathway remains uncertain [63].

More reflective of clinical disease, there is modeling evidence of cerebral microvascular injury in SARS-CoV-2–infected ACE2 transgenic mice in the absence of severe viral neuroinvasion [55, 64, 65]. Indeed, SARS-CoV-1–infected ACE2 transgenic mice have a lack of inflammation and astrocytic activation despite widespread neuronal infection [60]. Additionally, a recent in vitro study used induced pluripotent stem cells to derive brain endothelial cells and produced a Transwell model that was infected with the original strain of SARS-CoV-2. After infection, there was a decrease in transendothelial electrical resistance, claudin 3, and claudin 11, as well as a rise in the expression of proinflammatory genes [66].

## CLINICAL MANAGEMENT OF BBB DYSFUNCTION

There are limited established methods to manage BBB dysfunction clinically, although several approaches are under investigation (Table 1). The pathogenesis of the neuroinflammation leading to infection-driven BBB damage is multifactorial, depending on the etiology; therefore, initial management should aim to treat the infection, such as timely acyclovir for HSE [67]. For viral infections that do not have specific treatments (eg, JEV), vaccination should be encouraged for at-risk groups as primary prophylaxis. While respiratory COVID-19 management utilizes steroids, antivirals, and immune modulators, the research surrounding the management of neurologic complications is more limited [57, 68, 69, 71]. Nevertheless, the introduction of several treatments given primarily for respiratory disease, such as dexamethasone and remdesivir, has been associated with a decline in neurologic complications [68].

**Table 1.** Clinical and Preclinical Management Options for Blood-Brain Barrier Dysfunction

Clinical	Antivirals Examples: acyclovir for HSE and remdesivir for COVID-19 [67, 68] Prevention via vaccination Example: JEV vaccine Neuroprotective measures 30° head tilt CPP >60 or MAP >65 [69] Maintain electrolyte levels [70] Normothermia, normocapnia, normoglycemia [71] Osmotic therapies to control cerebral edema Hypertonic saline (superior for HSE) [72] Mannitol Corticosteroids Dexamethasone: DexEnceph trial [73] Steroids empirically used for COVID-19, but role in encephalopathy is limited in case reports [57, 68, 74, 75] ICP monitoring Invasive Noninvasive under exploration (eg, transcranial Doppler or optic nerve sheath diameter) [76, 77, 78]
Preclinical	Reducing MMP-9 activation with doxycycline limiting tight junction damage [58, 79, 80] MMP inhibition with batamistat reduced intracranial complications [81] Increasing GM130 expression reduced HSV-1 damage [82] Pan-caspase inhibitors showed a similar effect by increasing GM130 [82] CXCL-1 antagonism reduced HSE damage [7] AQP-4 channels could aid recovery for conditions with primarily vasogenic edema [58, 83] Increasing VE-cadherin with S1P reversed BBB permeability [84] S1P receptor modulators also shown to reduce damage [85] Neutrophil membrane nanoparticles identify the area of BBB inflammation [86] Thrombomodulin mRNA-loaded particles reduced inflammation of the endothelium [87] Resolvin D reduced leukocyte endothelial interaction [88]

Abbreviations: AQP-4, aquaporin 4; BBB, blood-brain barrier; CPP, cerebral perfusion pressure; CXCL-1, C-X-C motif chemokine ligand 1; GM130, Golgi matrix protein 130; HSE, herpes simplex encephalitis; HSV-1, herpes simplex virus 1; ICP, intracranial pressure; JEV, Japanese encephalitis virus; MAP, mean arterial pressure; MMP, matrix metalloproteinase; mRNA, messenger ribonucleic acid; S1P, sphingosine 1 phosphate; VE-cadherin, vascular endothelial cadherin.

### Cerebral Edema Management

The clinical management of BBB permeability during viral infection is often informed by research into traumatic brain injuries (TBIs). BBB damage can result in vasogenic edema from endothelial damage and reactive oxygen species, while direct toxic effects from pathogens can lead to cytotoxic edema [89]. Once damage to the BBB has occurred, neuroprotective measures should be taken to limit secondary brain insults, which could mediate further BBB breakdown and cerebral edema, causing diminished neurologic function, seizures, and increased intracranial pressure (reviewed by Cook et al [90]). Guidelines do not currently recommend primary seizure prophylaxis [69]. However, seizure risk stratification may inform the potential need for primary antiseizure prophylaxis [91].

Osmotic therapies such as mannitol and hypertonic saline are used in TBI to reduce cerebral edema and after BBB deterioration. A meta-analysis showed that osmotherapy improves outcomes when significant edema is present and found that hypertonic saline is superior to mannitol [72]. If the patient is hyponatremic, then mannitol may be safer due to the risk of central pontine myelinolysis with hypertonic saline. Evidence for osmotherapy in BBB dysfunction of an viral etiology is less robust, and no recommendations are agreed [69].

AQP-4 channels act as a passive pore, enabling the removal of extracellular fluid down pressure gradients [58]. Hence, these channels play a beneficial role in pathologies where vasogenic edema predominates, but they are detrimental when cytotoxic edema is present. For example, in a mouse model, significant downregulation of AQP-4 channels occurs in the acute phase of HSE with upregulation later in the disease process [83]. Thus, while modulation of AQP-4 channels may be beneficial, the timing in the disease process is crucial.

### Managing Inflammation and Prevention of Immune Cell Migration

Steroids are used for managing inflammation in many neurologic conditions. Dexamethasone increased the tightness between TJs, upregulated BBB Pgp (an efflux transporter), and in principle should mitigate BBB damage [92]. Steroids have evidence for treating meningitis, but the CRASH trial demonstrated an increase in mortality when used in the acute phase of TBI [92–94]. There is limited evidence for the use of steroids in viral encephalitis. While the use of steroids would likely aid in the management of edema, the effect on viral replication may be undesirable and is undetermined. The results of an adjunctive dexamethasone randomized controlled trial in HSV encephalitis in the United Kingdom are awaited [73]. There are minimal data regarding management of brain complications of COVID-19 steroids. Following the RECOVERY trial, steroids became a routine part of respiratory COVID-19 management for patients requiring oxygen [74]. Hence, steroids are commonly used for patients with COVID-19 encephalopathy/encephalitis, but there are no robust data supporting their use, beyond case series and observational studies [57, 75]. Since the COVID-19 pandemic, the incidence of neurologic cases has decreased, and treatment with dexamethasone has been found to be associated with fewer neurologic complications [68]. However, other contributing factors include protection by vaccines and evolution of SARS-CoV-2 variants [68].

The role of innate immune cells is another aspect of the pathophysiology that offers potential therapeutic targets. The transendothelial migration of neutrophils during HSE and other infectious encephalopathies is associated with increased morbidity, neurotoxicity, and BBB dysfunction via protease release and TJ disruption [7]. The infective process drives neuroimmune crosstalk with CXCL1 chemokine production by astrocytes and neurons in response to IL-1α. By blocking IL-1, the BBB damage is reduced, but the beneficial antiviral effects of IL-1 are also reduced. In human studies, the relative concentrations of the IL-1 family members to endogenous IL-1 antagonists is associated with cerebral edema and outcome [24]. As existing clinical therapies for IL-1 antagonism (IL-1RA) are in use for other conditions, this is an avenue for future investigation in HSE that may have implications for therapeutic application to other viral encephalitides.

Various studies have utilized nanoparticles derived from neutrophil membranes to maximize delivery to areas of inflammation within the BBB [86]. This was analyzed with thrombomodulin mRNA to protect the endothelial barrier from inflammation by inhibiting TNFα-induced BBB permeability [87]. A similar effect was found by using resolvin D, which decreased leukocyte–endothelial cell interaction [88]. However, the research into this delivery mechanism is minimal and has been limited to patients who experienced stroke.

### Modulation of Junction Complex Expression

The activation of MMPs in encephalitis and other cerebral insults is well documented and linked to BBB disruption via damage to TJ proteins [58], and inhibition of MMP activation was shown to significantly reduce this disruption [81]. Although MMPs may be harmful to the BBB during only the acute inflammatory phase, they inactivate cytokines while promoting angiogenesis and neurogenesis in the later phase [95]. Furthermore, much of the research on MMP inhibition is in the context of noninfectious cerebral injuries and may have undesirable effects on control of viral replication, particularly via proinflammatory cytokines. Moreover, MMPs can act as potent antiviral targets to control HSV-1 infection [82, 96]. Doxycycline may be a promising adjunct in reducing MMP-9 production [79, 80] and significantly reduces IL-1β–induced BBB injury by reducing damage to zonula occludens [97]. However, studies have examined the BBB only in the context of bacterial challenges.

Several studies have explored the localization of vascular endothelial cadherin, a key component in the adherent junctions [58, 98], as a method to reduce BBB permeability. The sphingolipid sphingosine 1 phosphate (S1P) maintains vascular endothelial cadherin at the endothelial cell-cell junctions [99]. Administering an S1P analogue has been shown to reverse BBB permeability in mice with preexisting BBB damage [84]. Additionally, a modulator of the S1P receptor (FTY720, fingolimod) prevented the redistribution of junctional proteins and ameliorated BBB damage and is used clinically for multiple sclerosis [85]. While FTY720 has beneficial effects in multiple sclerosis and encephalomyelitis, it has recently been shown to reactivate latent viral infections in murine models, thus necessitating additional research on the safety in virus-driven BBB damage [100].

A recent study of HSE noted the downregulation of occludin and claudin 5, affecting BBB integrity [82]. However, other possible mechanisms include downregulation of Golgi-associated protein GM130, Golgi fragmentation, and cell apoptosis. Overexpression of GM130 partially attenuated BBB damage. Furthermore, the use of a pan-caspase inhibitor, Z-VAD-fmk, led to increased levels of GM130, as well as occludin and claudin 5. This offers several potential targets for maintaining and restoring BBB functionality.

## CONCLUDING REMARKS

BBB injury is driven by neuroimmune crosstalk during viral infections and is crucial to the development of neurologic injury and sequelae. Current management strategies for virus-driven BBB dysfunction aim to treat the primary cause and prevent additional damage through neuroprotective measures, relying on findings primarily derived from TBI and stroke research. However, mortality and morbidity in viral-induced CNS injury remains high, and research is increasingly focused on preventing initial BBB permeabilization, limiting further BBB damage, and ultimately repairing the BBB. Future research should focus on delineating the neuroimmune pathways in cohorts with viral etiologies in conjunction with parallel in vivo and in vitro models to explore and determine optimal strategies that protect the brain.

## Supplementary Material

jiaf455_Supplementary_Data

## Supplementary Data

Supplementary materials are available at *The Journal of Infectious Diseases* online (http://jid.oxfordjournals.org/). Supplementary materials consist of data provided by the author that are published to benefit the reader. The posted materials are not copyedited. The contents of all supplementary data are the sole responsibility of the authors. Questions or messages regarding errors should be addressed to the author.
