# Supplementary material for: Viral Infection and the Blood-Brain Barrier: Molecular Research Insights and Therapies
Source: J Infect Dis. 2025 Sep 29;232(6):1273–82. doi: 10.1093/infdis/jiaf455 (PMC12718030; doi:10.1093/infdis/jiaf455)
Supplement: jiaf455_Supplementary_Data [file jiaf455_supplementary_data.docx]

Appendix

# ANATOMY & PHYSIOLOGY OF THE BBB

The BBB is comprised of brain endothelial cells (BECs), which form the lumen of blood vessels and are adjoined by characteristic tight junctions (TJs) and adherens junctions (AJs). The basement membrane underlying the endothelium is embedded within pericytes which partially encircle the BECs. The cerebral blood vasculature is encased by astrocytes with astrocytic endfeet projections. Adjacent cells found in the extravascular regions of the brain include neurons and microglia[1,2], which are considered part of the broader NVU (FIG. 1A). The highly specialised, restrictive regulatory properties of the BBB are unique to NVU and not developed in other organs. For example, transplantation of embryonic brain fragment grafts from quail endoderm tissue to chick non-neural tissue found in the avian coelomic cavity have been reported to vascularise with characteristics found within the BBB. Conversely, fragments of embryonic mesoderm transplanted into the brains of chick embryos do not induce BBB characteristic changes[3].

| **Component** | **Description** |
| --- | --- |
| Brain endothelial cells | TJs connect the plasma membrane of adjacent endothelial cells and consist of the transmembrane proteins claudins (claudin-3, -5, and -12), occludin, and junctional adhesion molecules (JAMs). In addition, cytoplasmic scaffolding proteins, known as zonula occludins-1, -2, -3 (ZO-1, ZO-2, ZO-3), link TJs to the intracellular actin cytoskeleton[4]. AJs form between adjacent BECs through complexes involving the transmembrane protein, E-cadherin, and the cytoplasmic protein, catenin, which also links to actin (FIG. 1B)[5].  Whilst oxygen and carbon dioxide can freely diffuse paracellularly from the bloodstream into the brain,[4] these TJs necessitate specific transport proteins for the passage of many important molecules across the BBB. Thus, only positively charged lipophilic molecules with a molecular weight <400 DA can freely diffuse across the BBB due to the expression of TJs and AJs[6]. Rather than simple diffusion, specifically regulated molecules must utilise carrier- (e.g., glucose, amino acids) or vesicular- (e.g., insulin, iron, leptin) mediated transcellular transport to traverse the BBB[4]. The up- or down-regulation of transmembrane proteins impact the integrity of the BBB. For example, claudin-5 is highly expressed in the endothelial cells in brain capillaries and is vital for barrier integrity. Mice deficient for claudin-5 display less restriction of larger molecules (<800 Da) across the BBB, allowing increased extravasation[7]. Conversely, another claudin, claudin-1, which is upregulated in mice and humans after a stroke, is associated with an increased BBB permeability and pro-inflammatory phenotype, with impaired recovery[8]. A decrease in the expression of claudin-5 also occurred with the increased presence of claudin-1. Vascular endothelial growth factor (VEGF) production by astrocytes has been linked to a down-regulation of both claudin-5 and occludin expression causing BBB breakdown[9]. VEGF production is significantly elevated in patients with encephalitis specifically caused by viral infection[10]. Other properties that allow BECs to be highly specialised for low permeability are a lack of fenestration, which reduces the number of areas where transcellular transport can occur, a lower rate of pinocytosis, and a higher number of mitochondria. More sophisticated *in vitro* modelling has implicated flow-based shear stress generated by cerebral blood flow leads to an increase in the expression of TJs, AJs and associated transcripts which maintain the low permeability of the BBB, which can be quantified *in vitro* by the transendothelial electrical resistance (TEER)[11,12]. However, it is impossible to know the full effects *in vivo* as a lack of cerebral blood flow would result in a non-viable model. Despite this, TEER values from *in vitro* models are shown to increase the most through the introduction of shear flow and can be comparable to *in vivo* TEER values[13,14] suggesting it is necessary for the development of mature barrier phenotypes. Additionally, the uptake of permeability dyes, such as Evans blue and Dextran, can be used to assess the solute permeability due to their different molecular sizes[15]. |
| Astrocytes | Astrocytes make up a significant portion of the cellular population in the CNS. The loss of astrocytes cannot be compensated for by other BBB components[16], thus they are essential for both BBB structure and function, as well as cell-to-cell communication between many of the NVU components, especially in response to inflammation[17,18]. In vitro work has shown that the presence of astrocytes increases the number of TJs[19] and likely further aid in the generation of specific barrier phenotype features. Astrocytes release a number of factors that maintain the integrity of the BBB, such as angiopoietin 1[20], basic fibroblast growth factor[21] and glial-derived neurotrophic factor[22], increasing the expression of TJs, and consequently the TEER[23,24]. Astrocytes grafted into capillaries with non-neural endothelial cells cause the capillaries to phenotypically change to become tighter and less permeable[25]. Aquaporin-4 (AQP4), found in astrocytic endfeet, regulates the transport of water across the BBB and this function is altered during systemic inflammation[26]^,^[27]. |
| Pericytes | Pericytes within the basement membrane partially encircle the capillary wall formed by BECs. Adhesion between pericytes and BECs is mediated through the protein N-cadherin. In the absence of N-cadherin, normal vascular morphogenesis cannot be achieved and this increases the permeability of the BBB[28,29]. Pericytes are important for the formation of TJs by inhibiting the expression of vascular molecules, vesicle trafficking[30], regulation of the entry of immune cells[31], angiogenesis, and control of blood flow through neurovascular coupling[32]. In pericyte-deficient mice, there is an increase in BBB permeability and abnormal polarization of astrocytic endfeet to the capillary[33]. Pericytes also show phagocytic properties and remove plasma proteins, such as immunoglobulins and albumin, in addition to cellular debris following brain injury[34]. |
| Microglia | Whilst not direct components of the BBB, but still part of the NVU, microglia can exert secondary effects on integrity of the BBB[35]. Microglia are the resident immune cells of the CNS and are established during embryonic development from yolk sac-derived precursors and are resident throughout life[36–38]. Microglia are extremely important in relation to surveillance of infectious pathogens in the brain, but also respond to systemic infection and can undergo phenotypic changes in the absence of CNS infection in response to systemic inflammation or neuronal injury[35,39]. Although there is no standardised morphological classification of microglia, commonly patrolling microglia are referred to as ‘resting’ or ‘ramified’, and glial cells that are highly branched, whereas during infection they may be considered as ‘activated’, ‘ameboid’ or ‘phagocytic’, with their appearance changing to become more rounded[40]. Other morphological states have been described (such as ‘rod-like’, ‘hyper-ramified’, and ‘ball-and-chain’) but these states lack consistent terminology, likely reflecting a continuum in degrees of activation making the use of the nomenclature to conclusively classify microglia challenging [41]. Importantly, the cytokines secreted by microglia can have downstream effects on other neuroglial cells, triggering a pro-inflammatory cascade that affects BBB function and integrity[35,42,43]. |
| Neurons | Neurons are cells that, through electrical excitation, transmit signals throughout the CNS controlling numerous cognitive, sensory, homeostatic, and movement functions. There are many different types of neurons which differ from region to region within the brain and also have different morphologies and connections related to their specialised function[44]. Neurons consist of a cell body, dendrites, an axon, and axon terminals. Similarly to microglia, neurons are also part of the NVU. The survival of neurons is dependent on an intact BBB as regeneration of neurons after damage from infection or injury is limited in humans, especially within the CNS[45]. Neurons can affect the BBB through the cytokines and chemokines that they secrete which attract neutrophils and other leukocytes into brain tissue[42]. Neurons can also regulate cerebral blood flow and BBB permeability through neurovascular coupling[46]. |

# REFERENCES

1. Griffin DE. Cytokines in the brain during viral infection: clues to HIV-associated dementia. J Clin Invest. **1997**; 100(12):2948–2951.

2. Chen J, Tan R, Mo Y, Zhang J. The blood-brain barrier in health, neurological diseases, and COVID-19. Fundam Res. **2022**; 2(5):817–826.

3. Stewart PA, Wiley MJ. Developing nervous tissue induces formation of blood-brain barrier characteristics in invading endothelial cells: A study using quail-chick transplantation chimeras. Dev Biol. **1981**; 84(1):183–192.

4. Kadry H, Noorani B, Cucullo L, Luca Cucullo. A blood–brain barrier overview on structure, function, impairment, and biomarkers of integrity. Fluids Barriers CNS. **2020**; 17(1):1–24.

5. Hartsock A, Nelson WJ. Adherens and Tight Junctions: Structure, Function and Connections to the Actin Cytoskeleton. Biochim Biophys Acta. **2008**; 1778(3):660–669.

6. Bellettato CM, Scarpa M. Possible strategies to cross the blood–brain barrier. Ital J Pediatr. **2018**; 44(2):131.

7. Ohtsuki S, Sato S, Yamaguchi H, Kamoi M, Asashima T, Terasaki T. Exogenous expression of claudin-5 induces barrier properties in cultured rat brain capillary endothelial cells. J Cell Physiol. **2007**; 210(1):81–86.

8. Sladojevic N, Stamatovic SM, Johnson AM, et al. Claudin-1-Dependent Destabilization of the Blood–Brain Barrier in Chronic Stroke. J Neurosci. **2019**; 39(4):743–757.

9. Argaw AT, Gurfein BT, Zhang Y, Zameer A, John GR. VEGF-mediated disruption of endothelial CLN-5 promotes blood-brain barrier breakdown. Proc Natl Acad Sci. Proceedings of the National Academy of Sciences; **2009**; 106(6):1977–1982.

10. Mori D, Khanam W, Sheikh RA, et al. Increased serum vascular endothelial growth factor is associated with acute viral encephalitis in Bangladeshi children. Sci Rep. **2017**; 7(1):16181.

11. Cucullo L, Hossain M, Puvenna V, Marchi N, Janigro D. The role of shear stress in Blood-Brain Barrier endothelial physiology. BMC Neurosci. **2011**; 12:40.

12. Patabendige A, Janigro D. The role of the blood–brain barrier during neurological disease and infection. Biochem Soc Trans. **2023**; 51(2):613–626.

13. Weksler B, Romero IA, Couraud P-O. The hCMEC/D3 cell line as a model of the human blood brain barrier. Fluids Barriers CNS. **2013**; 10(1):16.

14. Cucullo L, Couraud P-O, Weksler B, et al. Immortalized Human Brain Endothelial Cells and Flow-Based Vascular Modeling: A Marriage of Convenience for Rational Neurovascular Studies. J Cereb Blood Flow Metab. SAGE Publications Ltd STM; **2008**; 28(2):312–328.

15. Fu BM, Zhao Z, Zhu D. Blood-Brain Barrier (BBB) Permeability and Transport Measurement In Vitro and In Vivo. Methods Mol Biol Clifton NJ. **2021**; 2367:105–122.

16. Heithoff BP, George KK, Phares AN, Zuidhoek IA, Munoz-Ballester C, Robel S. Astrocytes are necessary for blood–brain barrier maintenance in the adult mouse brain. Glia. **2021**; 69(2):436–472.

17. Abbott NJ, Rönnbäck L, Hansson E. Astrocyte-endothelial interactions at the blood-brain barrier. Nat Rev Neurosci. **2006**; 7(1):41–53.

18. Huang L, Nakamura Y, Lo EH, Hayakawa K. Astrocyte Signaling in the Neurovascular Unit After Central Nervous System Injury. Int J Mol Sci. **2019**; 20(2):282.

19. Tao-Cheng J, Nagy Z, Brightman M. Tight junctions of brain endothelium in vitro are enhanced by astroglia. J Neurosci. **1987**; 7(10):3293–3299.

20. Lee S-W, Kim WJ, Choi YK, et al. SSeCKS regulates angiogenesis and tight junction formation in blood-brain barrier. Nat Med. Nature Publishing Group; **2003**; 9(7):900–906.

21. Huang B, Krafft PR, Ma Q, et al. Fibroblast growth factors preserve blood-brain barrier integrity through RhoA inhibition after intracerebral hemorrhage in mice. Neurobiol Dis. **2012**; 46(1):204–214.

22. Igarashi Y, Utsumi H, Chiba H, et al. Glial cell line-derived neurotrophic factor induces barrier function of endothelial cells forming the blood-brain barrier. Biochem Biophys Res Commun. **1999**; 261(1):108–112.

23. Haseloff RF, Blasig IE, Bauer HC, Bauer H. In search of the astrocytic factor(s) modulating blood-brain barrier functions in brain capillary endothelial cells in vitro. Cell Mol Neurobiol. **2005**; 25(1):25–39.

24. Neuhaus J, Risau W, Wolburg H. Induction of Blood-Brain Barrier Characteristics in Bovine Brain Endothelial Cells by Rat Astroglial Cells in Transfilter Coculturea. Ann N Y Acad Sci. **1991**; 633(1):578–580.

25. Janzer RC, Raff MC. Astrocytes induce blood–brain barrier properties in endothelial cells. Nature. Nature Publishing Group; **1987**; 325(6101):253–257.

26. Ja H, Ji S, Dk B. The role of aquaporin-4 in synaptic plasticity, memory and disease. Brain Res Bull [Internet]. Brain Res Bull; **2018** [cited 2023 Oct 5]; 136. Available from: https://pubmed.ncbi.nlm.nih.gov/28274814/?dopt=Abstract

27. Díaz-Castro B, Robel S, Mishra A. Astrocyte Endfeet in Brain Function and Pathology: Open Questions. Annu Rev Neurosci. **2023**; 46:101–121.

28. Gerhardt H, Wolburg H, Redies C. N-cadherin mediates pericytic-endothelial interaction during brain angiogenesis in the chicken. Dev Dyn Off Publ Am Assoc Anat. **2000**; 218(3):472–479.

29. Kruse K, Lee QS, Sun Y, et al. N-cadherin signaling via Trio assembles adherens junctions to restrict endothelial permeability. J Cell Biol. **2019**; 218(1):299–316.

30. Daneman R, Zhou L, Kebede AA, Barres BA. Pericytes are required for blood–brain barrier integrity during embryogenesis. Nature. Nature Publishing Group; **2010**; 468(7323):562–566.

31. Pieper C, Marek JJ, Unterberg M, Schwerdtle T, Galla H-J. Brain capillary pericytes contribute to the immune defense in response to cytokines or LPS in vitro. Brain Res. **2014**; 1550:1–8.

32. Hall CN, Reynell C, Gesslein B, et al. Capillary pericytes regulate cerebral blood flow in health and disease. Nature. **2014**; 508(7494):55–60.

33. Armulik A, Genové G, Mäe M, et al. Pericytes regulate the blood–brain barrier. Nature. Nature Publishing Group; **2010**; 468(7323):557–561.

34. Winkler EA, Sagare AP, Zlokovic BV. The Pericyte: A Forgotten Cell Type with Important Implications for Alzheimer’s Disease? Brain Pathol. **2014**; 24(4):371–386.

35. Haruwaka K, Ikegami A, Tachibana Y, et al. Dual microglia effects on blood brain barrier permeability induced by systemic inflammation. Nat Commun. Nature Publishing Group; **2019**; 10(1):5816.

36. Ginhoux F, Greter M, Leboeuf M, et al. Fate Mapping Analysis Reveals That Adult Microglia Derive from Primitive Macrophages. Science. **2010**; 330(6005):841–845.

37. Ginhoux F, Prinz M. Origin of Microglia: Current Concepts and Past Controversies. Cold Spring Harb Perspect Biol. **2015**; 7(8):a020537.

38. Kierdorf K, Erny D, Goldmann T, et al. Microglia emerge from erythromyeloid precursors via Pu.1- and Irf8-dependent pathways. Nat Neurosci. **2013**; 16(3):273–280.

39. Zrzavy T, Höftberger R, Berger T, et al. Pro-inflammatory activation of microglia in the brain of patients with sepsis. Neuropathol Appl Neurobiol. **2019**; 45(3):278–290.

40. Woodburn SC, Bollinger JL, Wohleb ES. The semantics of microglia activation: neuroinflammation, homeostasis, and stress. J Neuroinflammation. **2021**; 18(1):258.

41. Vidal-Itriago A, Radford RAW, Aramideh JA, et al. Microglia morphophysiological diversity and its implications for the CNS. Front Immunol. **2022**; 13:997786.

42. Michael BD, Bricio-Moreno L, Sorensen EW, et al. Astrocyte- and Neuron-Derived CXCL1 Drives Neutrophil Transmigration and Blood-Brain Barrier Permeability in Viral Encephalitis. Cell Rep. **2020**; 32(11):108150.

43. Shigemoto-Mogami Y, Hoshikawa K, Sato K. Activated Microglia Disrupt the Blood-Brain Barrier and Induce Chemokines and Cytokines in a Rat in vitro Model. Front Cell Neurosci. **2018**; 12:494.

44. Banerjee S, Bhat MA. Neuron-Glial Interactions in Blood-Brain Barrier Formation. Annu Rev Neurosci. **2007**; 30:235–258.

45. Steward MM, Sridhar A, Meyer JS. Neural Regeneration. In: Heber-Katz E, Stocum DL, editors. New Perspect Regen [Internet]. Berlin, Heidelberg: Springer; 2013 [cited 2023 Apr 4]. p. 163–191. Available from: https://doi.org/10.1007/82_2012_302

46. Kaplan L, Chow BW, Gu C. Neuronal regulation of the blood–brain barrier and neurovascular coupling. Nat Rev Neurosci. Nature Publishing Group; **2020**; 21(8):416–432.
